# Advanced Periocular Basal Cell Carcinoma with Orbital Invasion: Update on Management and Treatment Advances

**DOI:** 10.1155/2024/4347707

**Published:** 2024-02-29

**Authors:** Alvaro Bengoa-González, Enrique Mencía-Gutiérrez, María Garrido, Elena Salvador, María-Dolores Lago-Llinás

**Affiliations:** ^1^Ophthalmology Department, 12 de Octubre Hospital, Complutense University, Madrid, Spain; ^2^Pathology Department, 12 de Octubre Hospital, Complutense University, Madrid, Spain; ^3^Radiology Department, 12 de Octubre Hospital, Complutense University, Madrid, Spain

## Abstract

**Purpose:**

Basal cell carcinoma (BCC) is the most frequent malignant periocular tumor. It is associated with exposure to ultraviolet radiation, and its incidence is gradually increasing. It may occasionally display more aggressive behavior and result in orbital or intracranial invasion. Mortality from periocular BBC with orbital invasion is very low, but the associated morbidity can be significant, from disfigurement to blindness. Traditionally, these cases have been treated with orbital exenteration or with radiotherapy (RT), but in recent years, hedgehog pathway inhibitors (HPIs) have emerged, are effective in more serious cases, and are used primarily or combined with surgery, changing our perspective on the management of these patients.

**Methods:**

We studied 24 cases of periocular BCC with orbital invasion, some primary and others recurrent, which were treated between 2011 and 2021 in the same hospital. All patients had clinical or radiological evidence of orbital invasion. Orbital exenteration was performed on 9/24 of the patients (1 received vismodegib after surgery), and 12/24 were treated, surgically preserving the eyeball, with 3 of them receiving adjuvant vismodegib. Three of the twenty-four patients were treated exclusively with vismodegib (Erivedge®, Genentech).

**Results:**

One patient died due to poor tumor evolution, but the rest evolved favorably and they have had no recurrences. Vismodegib was generally well tolerated, except for in one patient who discontinued treatment due to the side effects.

**Conclusions:**

In advanced BBC with orbital invasion, mutilating surgical treatments such as exenteration or potentially vision-threatening treatments such as RT remain as options. In recent years, however, very promising new medical therapies have emerged, such as HPI, which can be used effectively instead of surgery or in combination with it, preserving the eye and vision, which implies a new approach to treatment.

## 1. Introduction

Basal cell carcinoma (BCC) is the most common skin cancer. Seventy-five per cent of these cancers occur in the head and neck and around 20% in the periocular region [1]. It is the most common periocular malignant tumor, approximately 90% of cases, and its incidence is gradually increasing [2]. It usually occurs in older people and with exposure to ultraviolet radiation (UVR). One of the most important risk factors is intense and intermittent exposure to UVR or long-term abuse of sun exposure, since it can damage the deoxyribonucleic acid (DNA) and its repair system, causing mutations (carcinogenesis) that will be responsible for the abnormal activation of sonic hedgehog (Hh) pathways, causing uncontrolled cell activation and proliferation [3].

In most cases, the tumor is cured with local surgical excision but, occasionally, it may show more aggressive behavior and result in orbital or intracranial invasion [4].

Orbital invasion by periorbital BCC is uncommon, with reported incidences of 0.8–5.5% [5] in developed countries and even 17% in developing countries [6]. It can often be clinically silent, which makes it necessary to be alert to high risk tumors [7]. Risk factors include multiple recurrences, large tumor size, positive surgical margins, aggressive histologic subtype (morpheiform/sclerosing, infiltrative, basosquamous cell), location in the medial or lateral canthal area, advanced age of the patient and perineural invasion (PNI), present in less than 1% of cases [5, 8–11].

Although mortality from periocular BCC with orbital invasion is very low, associated morbidity can be significant, from disfigurement to blindness [4, 7, 12].

Orbital invasion by BCC has traditionally been managed by exenteration, which can be very disfiguring, as well as entailing significant surgical and psychological morbidity due to loss of the eyeball. Another treatment, radiation therapy (RT), is less effective than surgery and carries considerable risk of ocular toxicity [8, 13, 14]. In select cases with anterior orbital invasion, conservative tumor excision preserving the eyeball, with or without adjuvant RT, has proven to be pathway a viable alternative [8, 9, 11].

In recent years, we also have HPI, vismodegib (Erivedge®, Genentech) [15] and sonidegib (Odomzo®, Novartis) [16], to treat locally advanced carcinomas that have recurred after surgery, or which are not candidates for surgery or RT, or for cases with multiple lesions with BCC as in Gorlin-Goltz syndrome; these can be used as the main treatment or combined with surgery [6, 15–19].

We describe a series of 24 patients with histologically confirmed, orbital invasion by BCC who were managed with exenteration, conservative (nonexenterating) surgery, or vismodegib, as a sole treatment or adjuvant after local or radical excision. We analyze the clinical presentations, indications for surgery, surgical techniques, alternatives to radical surgical treatment, and new approaches in reference to advanced periocular BCC with orbital invasion.

## 2. Materials and Methods

We present a retrospective study of 24 cases of BCC with orbital invasion of 487 eyelid or periocular BCC, referred and treated in the Oculoplastic Unit of the 12 de Octubre University Hospital, Madrid, Spain, between January 2011 and February 2021. Each lesion has been coded according to the cancer staging manual for carcinoma of the eyelid by the American Joint Committee on Cancer (AJCC) 8^th^ edition [20].

The inclusion criteria were histologically confirmed BCC, recurrent or primary, of the medial or lateral canthal region, in the lower or upper eyelid and with clinical suspicion, radiological or histological evidence of orbital invasion. An imaging test, a computed tomography (CT) scan of the orbits or a magnetic resonance imaging (MRI) scan, was performed to confirm orbital invasion, by infiltration of soft tissues, involvement or not of the sclera and extraocular muscles, and bone infiltration or adjacent cavities. Those patients whose diagnosis could not be confirmed clinically, radiologically, or histologically were excluded, as were all those patients with orbital invasion who declined any therapeutic option.

Twenty-one of the twenty-four cases were managed by excision with margin control; they were all histologically studied. In 18/24 cases, the histological analysis was done through sections with paraffin, and in 6/24 with standard frozen section.

The treatments proposed for each patient were chosen based on the size of the tumor and the extension in the orbit, the number of recurrences and previous treatments, age, general condition of the patient, vision, and final decision of the patient among the therapeutic options offered.

All surgeries were performed by the same senior surgeon (A.B–G). All patients were evaluated at follow-up by the same oculoplastic surgeons and neuroradiologists. The oculoplastic surgeon closely monitored each postoperative case.

Patients treated with vismodegib alone underwent monthly assessments, blood tests, and monitoring of kidney and liver function. Radiology assessments were performed every 4 months during the first year and at intervals thereafter. Progression or recurrence was determined based on clinical and radiologic findings. Clinical responses were assessed according to what was previously reported in the literature [6]. Complete tumor remission was defined as the absence of clinical and radiologic signs suspicious for tumor, as determined by biopsy if needed.

Data were collected on demographic characteristics; location, tumor presentation, histological characteristics and therapeutics options, as well as the type of surgical intervention with or without adjuvant treatment and follow-up (Table 1).

This work was carried out following the recommendations of the Declaration of Helsinki of 1964 (last amendment, 2013), and it has the approval of the 12 de Octubre University Hospital Research Committee and Clinical Research Ethics Committee. In addition, the patients signed an informed consent for surgery and/or for the treatment with vismodegib. There is no conflict of interest and there is no funding from public or private organizations.

## 3. Results

Twenty-four patients were included, 13 males and 11 females, with a mean age of 72 years (range 42–95 years) with periocular BCC with invasion of adjacent ocular or orbital structures through the septum, classified as T4 according to the AJCC 8^th^ edition criteria for eyelid carcinoma [20]. The tumors were recurrent in 70.83% (17/24) of the cases; that is, they had been previously operated on in the same area or were treated with RT. In 14/17 cases, no tumor-free margins had been obtained. The mean time from initial surgery to recurrent disease and orbital involvement was 48.94 months (4.07 years), ranging from 15 to 96 months. Seven cases were primary tumors, meaning that there may or may not have been BCC elsewhere on the body or face, but not in the periocular area, prior to diagnosis.

**Figure 1:**
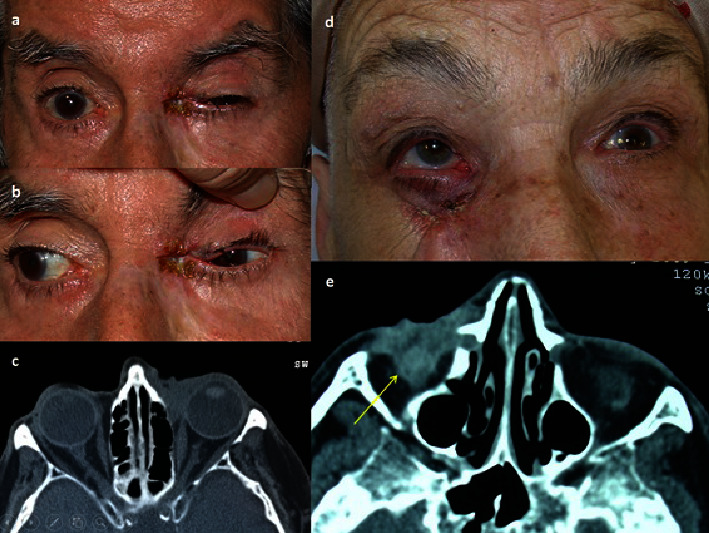
Recurrent basal cell carcinoma (BCC) (patient 11 in Table 1). (a) Ulcerative lesion in the medial canthus, adherent to deep planes. (b) Contraction of the eyelids that makes them difficult to open. Limitation in abduction of the eyeball. (c) Axial computed tomography scan demonstrates a soft tissue mass in the left inner of canthus of left orbit. The lesion shows homogeneous enhancement with slightly regular borders. The mass infiltrates the skin and subcutaneous layers, at the level of the inferior eyelid, involving the medial corner of the eye. There is infiltration of the medial inferior tarsal plate, of the anterior orbital tissue, and the anterior portion of the medial rectus muscle. There is no fatty plane of separation with the sclera (arrow). (d) Recurrent BCC (patient 7). Ulcerated mass in the lower periocular region, adherent to deep planes, and causing an upward displacement of the eyeball. (e) Radiographic confirmation of an irregular mass infiltrating the inferior orbital tissue, inferior rectus muscle, and orbital floor (arrow).

**Figure 2:**
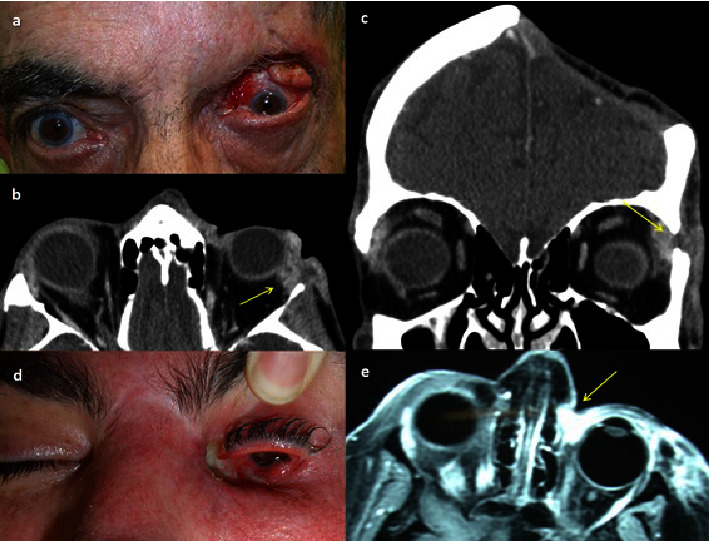
(a) Recurrent basal cell carcinoma (BCC) in upper eyelid treated with radiotherapy (RT) (patient 4). Ulcerated mass that has eroded upper eyelid and periocular tissue up to the orbital roof, adherent to deep planes, and with downward displacement of the eyeball. (b) Axial computed tomography (CT) scan demonstrates a soft tissue mass in the left outer canthus of the left orbit. The lesion shows mild enhancement with irregular borders. Skin is infiltrated at the level of the superior eyelid with lacrimal gland invasion (arrow). (c) There is bone destruction of the lateral margin of the roof (arrow) (patient 4). There is no fatty plane of separation with the sclera and the anterior portion of the lateral rectus muscle. (d) Recurrent BCC with large ulceration in the medial canthus and part of the lower eyelid (patient 1). Limitation of ocular motility. (e) CT scan showing infiltration of the anterior orbit, lacrimal and ethmoid bones, sclera, and anterior portion of the medial rectus muscle (arrow).

In almost all patients (22/24) we find as the most frequent sign, when of orbital invasion was suspected, a palpable mass or a visible ulcerated lesion (Figures 1(a), 1(d), 2(a), and 2(d)). Eyelid retraction was present in 8/24 patients and was the predominant sign in 2/24 cases (Figures 1(d), 2(a), and 2(d)). In 10/24 patients, adherence to deep planes and to the bone was suspected on palpation. In 12/24 cases, there was limitation in ocular motility and in 8/24 displacement of the eyeball (Figures 1(b), 1(d), 2(a), and 2(d)). In one patient, the mass completely covered the periocular and eyelid area, preventing examination of the eye.

**Figure 3:**
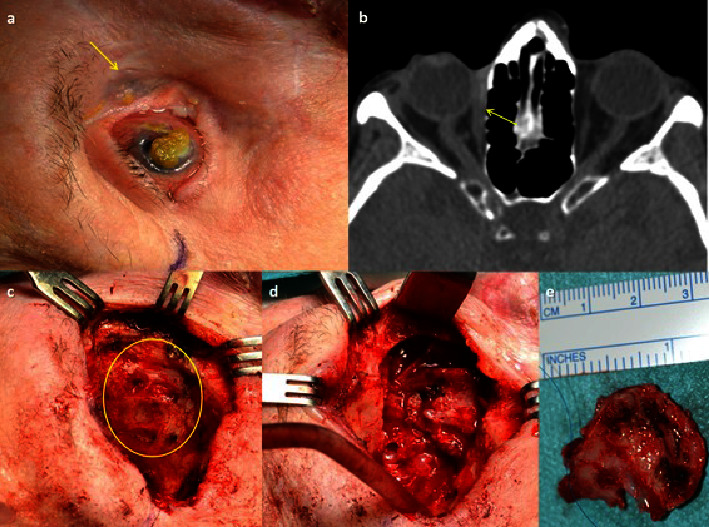
(a) Recurrent basal cell carcinoma (patient 15), treated with surgery and RT. Severely damaged ocular surface due to RT. Dark colored mass adherent to deep planes and limitation of ocular motility (arrow). (b) Computed axial tomography shows a soft tissue mass in the right medial canthus of the orbit. It presents a slight contrast uptake with irregular margins in the medial orbital space. The lesion produces a loss of continuity of the insertion of the internal rectus muscle (arrow). Suspicion of cortical bone involvement. (c–e). After exenteration, multiples areas of bone erosion in the orbital medial wall are found (circle), which is extracted in bloc for histopathological study that will confirm its infiltration by the tumor.

**Figure 4:**
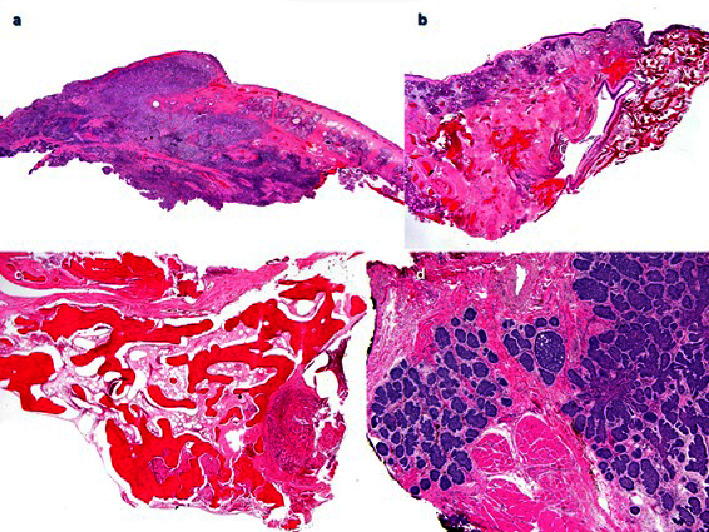
(a) Infiltrating basal cell carcinoma (BCC) showing deep invasion of periocular soft tissue close (H&E staining, ×10). (b) Periocular infiltrating BCC showing deep invasion of periocular soft tissue close to the lacrimal duct (H&E staining, ×10). (c) Periocular infiltrating BCC showing invasion of the orbital bone (H&E staining, ×10). (d) Infiltrative BCC with micronodular pattern showing deep invasion of the eyelid and periorbital soft tissue (H&E staining, ×10).

A radiological test was performed (CT or MRI) in all patients, confirming the orbital invasion in 18/22 cases (Figures 1(c), 1(e), 2(b), 2(c), 2(e), and 3(b)), and in 7/22 cases, invasion of bone walls was observed. In questionable radiological cases, orbital invasion was confirmed by histological study after surgery (Figures 3(a)–3(d) and 4(c)).

The most common histologic pattern was infiltrative (22/24), with one basosquamous and one micronodular case (Figures 4(a) and 4(d)). While 66.6% of the tumors (16/24) involved the medial canthus region (one of them in addition to the lower eyelid), 12.5% (3/24) affected the lateral canthus (one of them with extension to the upper eyelid), and the remainder 16.66%, were located in the lower eyelid (4/24), and one case completely covered the eyelids and the periocular area.

### 3.1. Treatment

In 12/24 cases, conservative surgical management was performed with globe preservation. Delayed reconstruction was performed 72 hours after excision of the tumor, once it was confirmed that the margins were negative. If we could not achieve tumor-free margins, we performed the necessary studies until we did and reconstructed the defect in the following days. In 3/12 cases, tumor-free margins could not be achieved without damaging orbital or ocular structures, and adjuvant vismodegib was added for several months (Table 1).

Orbital exenteration was performed in 9 cases (6 of them before 2016), one of them after conservative surgical treatment and prior RT in another center. In 7/9 cases, in addition to extension to the soft tissues, there was extension of the tumor to the orbital bone walls (Figures 3(a)–3(d)). In 2 patients, histologically positive tumor cells were detected in the paranasal sinuses, so adjuvant treatment was added after surgery. Case 1 in Table 1 received 60 Gy postoperative RT to the cavity and sinus (as vismodegib was not yet available), and case 15 received 150 mg vismodegib daily for 8 months postoperatively.

**Figure 5:**
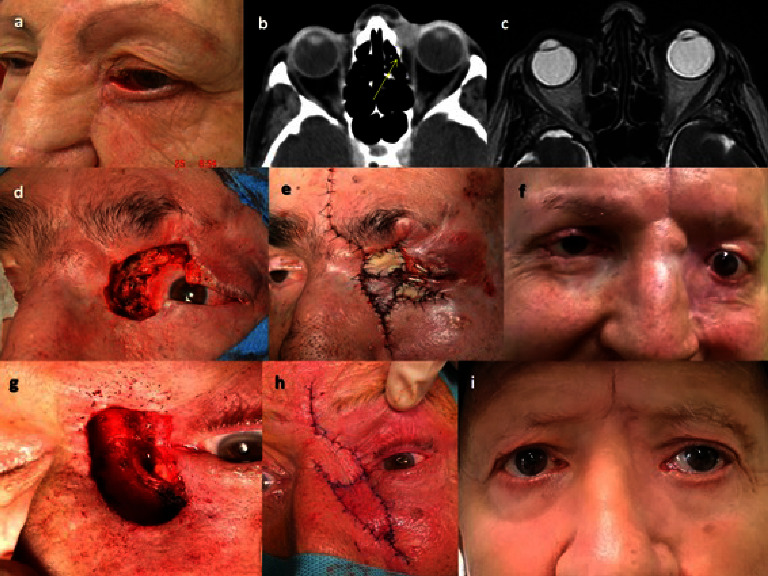
(a) Recurrent basal cell carcinoma (BCC) in medial canthus (patient 9). Mass adherent to deep plane that limits ocular motility. (b) Computed tomography scan confirms infiltration of the anterior orbit and anterior portion of the medial rectus muscle. (c) Nuclear magnetic resonance shows complete disappearance of the mass following treatment only with vismodegib (patient 9). (d) Recurrent BCC (patient 16) in medial canthus in a patient with Gorlin's syndrome with infiltration of the anterior orbital tissue. Treatment consisted in removal of the tumor maintaining the eyeball, with infiltrated margins, and subsequent treatment with vismodegib. (e) Reconstruction with flap and grafts. (f) Good result and no recurrence after treatment with vismodegib. (g) Recurrent micronodular basal cell carcinoma infiltrating anterior orbital tissue and lacrimal sac (patient 24). Treatment consisted in extirpation of the mass and lacrimal sac, maintaining the eyeball. (h) Reconstruction combining island pedicle flap and glabellar flap. Subsequent treatment with vismodegib after surgery due to infiltrated margins. (i) Good cosmetic result and no recurrences after surgery.

In 3/24 cases, both surgery and RT were refused, excluding vismodegib as the only treatment (post-2016) (Figures 5(a)–5(c)). The regimen was 150 mg daily for 12 months in one of them (case 9 in table), 3 months in another case (22) where the patient had to discontinue treatment while receiving another treatment for lung cancer, and 1 month in another patient (case 23) who stopped treatment due to intolerance of side effects and age.

Mean vismodegib treatment after surgery was 21.5 months, range 1–58 months. The dose was 150 mg per day. In one of the cases, there were 2 treatment breaks of 2 months each during the course of treatment.

Muscle cramps were the most common side effect (5/7), followed by dysgeusia (4/7), alopecia (3/7), weight loss (3/7), and gastric discomfort (3/7). Patient 16, with Gorlin's syndrome, received adjuvant vismodegib for 42 consecutive months after surgery with good tolerability. However, due to increased side effects, the regimen was subsequently modified with a 2-month break each year with better tolerability and no relapses.

### 3.2. Surgical Techniques

#### 3.2.1. Orbital Exenteration

**Figure 6:**
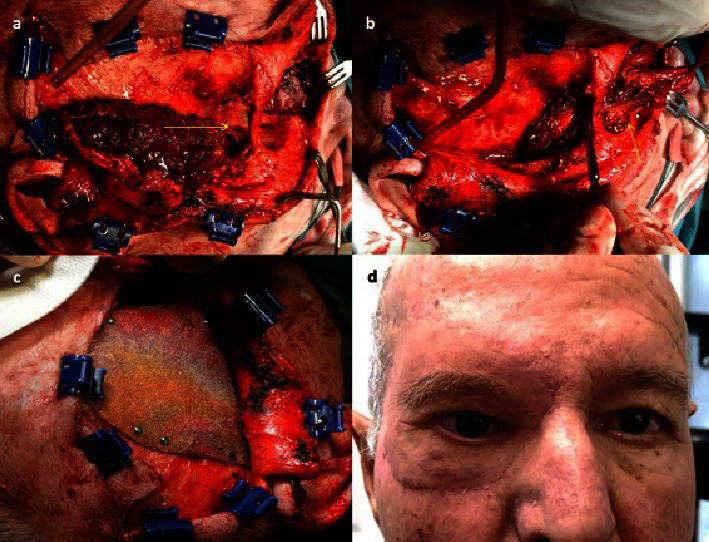
(a) The temporalis muscle is uninserted through a hemicoronal approach. A bone window is performed extending from the frontozigomatic fissure to the orbital floor, resecting the deep lateral wall (arrow). (b) The temporalis muscle flap is passed through the bone window to cover the orbital cavity. (c) A Medpor® prosthesis is placed in the temporal fossa and fixed with titanium screws to prevent temporal hollowing. (d) Acceptable cosmetic result after fixing an oculofacial prosthesis.

Two types of orbital exenteration were performed: removing soft tissue without removing bone walls and extended exenteration with bone walls, preserving or not eyelid skin. We performed exenteration extended to bone walls in 7/9 cases, performing ethmoidectomy in 4 of them (Figures 3(c)–3(e)), maxillectomy in 1 case, ethmoidectomy plus maxillectomy in 1 case and orbital lateral wall plus orbital roof in another. In all cases, the orbital cavity was covered with a transposition of a temporalis muscle flap using a transorbital approach. In 6/9 cases, a Medpor® implant (Stryker, Kalamazoo, MI) was also placed in the temporal fossa to resolve the hollowing caused (Figures 6(a)–6(c)). In cases where the carcinoma extended to the bone, the resection was performed with a 4-5 mm margin, eliminating it in bloc, placing a titanium mesh in place, and finally covering it with the temporalis muscle. The skin defects were covered with the healthy skin of the eyelids, when it could be preserved or with skin flaps from adjacent areas. When it was not possible to use flaps from neighboring sites, full-thickness skin grafts from other donor sites, such as the inner arm, were used.

#### 3.2.2. Globe-Sparing Surgery

In cases of conservative surgery preserving the eyeball (12/24), excision of the tumor was performed with a 4-5 mm margin subsequently, confirming that these were negative for cancer cells. After 72 hours, the resulting defects were reconstructed using complex oculoplastic reconstruction techniques, with skin flaps from nearby areas or combined with grafts. The island pedicled flap for reconstruction of defects in the medial canthal was the most widely use flap, alone (3/12) or combined with other flaps (6/12) (Figures 5(e) and 5(h)). The glabellar flap was another of the most commonly used flaps, always in combination with other flaps (4/12); in 2/12 cases, both flaps were combined. The tarsoconjunctival flap, associated or not with a periosteal flap, was the most widely used for reconstruction of the posterior lamella of the eyelid, combined with skin flaps (glabellar/Mustardé) or full-thickness skin grafts, generally obtained from the retroauricular area, for the reconstruction of the anterior lamella. In the patient with Gorlin's syndrome and recurrent cancer in the area of the medial canthus and upper eyelid, previously operated with a glabellar flap, the resulting defect in the canthal area was covered with a pericranial flap to vascularize the defect bed, in addition to a pedicled island flap, along with an advanced upper eyelid tarsoconjunctival flap to reconstruct the upper eyelid defect, plus skin grafts from the retroauricular area. (Figures 5(d)–5(f)) (Table 1).

In 4/12 cases, exeresis of infiltrated bone with a 4-5 mm margin was also performed, followed by placement of a titanium mesh. In 6/12 cases, exeresis of the lacrimal sac was performed due to suspected invasion of the lacrimonasal duct, with the invasion being confirmed in 4 patients (Figures 4(b) and 5(g)).

### 3.3. Follow-Up

Mean follow-up was 52 months with a range of 8–76 months. There were 12/24 patients who were followed for at least 60 months. The remaining cases did not reach 60 months of follow-up due to recent treatment or other reasons such as advanced age, discontinuation of treatment, loss to follow-up due to geographic distance and death.

Eight of the nine patients who underwent of orbital exenteration did not suffer recurrence during the follow-up period. Despite treatment (exenteration and RT), patient 1 died of the tumor almost 4 years after orbital invasion due to extension into the paranasal sinuses (subsequently operated on by maxillofacial surgeons) and the cranial fossa.

Twelve of the twenty-four cases that received conservative treatment of the eyeball, with or without adjuvant treatment, have had no recurrences to date.

Overall response to vismodegib was based on clinical and radiologic evaluation using the Response Evaluation Criteria for Solid Tumors (RECIST) for primary or adjuvant therapy. Patient 9 in Table 1, treated with vismodegib alone, had complete tumor clearance at 8 months with no radiological evidence of residual disease. It was decided to continue treatment for several more months. Patient 22 was treated with vismodegib for 3 months before discontinuation due to treatment of another tumor. He showed significant improvement in all of his lesions, but follow-up was inadequate due to his systemic deterioration. Patient 23 stopped treatment one month after starting due to side effects.

To date, no patient treated with adjuvant vismodegib after surgery has shown tumor recurrence during the follow-up period. Eight patients died during follow-up due to causes unrelated to the BCC, except for one as noted above.

Epiphora was the most frequent sequel in occurring in 8/12 patients. Other sequelae that were observed were diplopia (2/12), paresthesia in the orbital area (5/12), and those derived from reconstruction techniques such as skin graft necrosis (1/24) and eyelid retraction after surgery (Table 1).

## 4. Discussion

Orbital invasion occurs infrequently in BCC, but when it occurs, it increases the morbidity rate and lowers the cure rate, and can lead to disfigurement or blindness, and even death [6]. In these cases, a combination of various therapies, surgical, RT, and medical, may be required to achieve the adequate response [21].

### 4.1. Risk Factors in Advanced Periocular BCC

The series of patients with advanced BCC with orbital invasion that we present is one of the largest coming from the same center, where we have been able to analyze the important evolution of the management of these cases in recent years. We have verified in our study a low incidence of this pathology, less than 5%, 4.92% (24/487) of cases in a single oculoplastic surgery unit, similar to what has been reported in other studies [21], although an incidence of 17% has been reported [22].

The demographics of our study are in line with those observed in other studies, in terms of the age of the patients (>70 years) and the preponderance of males (62.5%) [8, 9, 12, 15].

BCC is a malignant tumor that, when located in the periocular region and invading the orbital tissues, is associated with a worse prognosis, because it can potentially lead to intracranial invasion and death [8, 9]. Some authors [23] indicated years ago that an incomplete excision can contribute to the development of a more aggressive tumor, since the fibrosis caused by the scar can prevent malignant cells from migrating to the surface, therefore favoring its extension in depth, hindering its control, and delaying clinical detection of recurrence (Figures 3(a), 5(d), and 5(g)). Previous recurrence is the main risk factor in subsequent recurrences, increasing by more than 50% after a third intervention [11, 12, 24]. Most of the cases with orbital invasion in our work were recurrent tumors (70.8%), due to incomplete excisions or previous nonsurgical treatments that prevented negative margins from being verified, as occurred in 82.3% (14/17) of them.

All of the cases that we report correspond to the most aggressive histological types (infiltrative 22/24, micronodular, and basosquamous), where it is more difficult to obtain clear margins after surgical excision, in addition to implying greater morbidity in relation to its surgical management, as has been indicated in other works [8, 9, 25]. The morpheiform histological type has not been included. The histological explanation is that the morphean BCC is characterized by its prominent stromal fibrosis accompanying the epithelial elements of thin strands and small nests of neoplastic cells with only limited peripheral palisading and peritumoral clefts. It has many synonyms including fibrosing, sclerosing, and syringomatous BCC. A related variant is infiltrative BCC, which presents with a rather looser mucinous stroma, although most tumors combine features of both and they are all included under the wider name of infiltrative BCC [26]. The infiltrative growth pattern is the most frequent observed in cases of orbital invasion (81.8% in Howard et al.) [12], reaching 91% in our study.

Extratumoral PNI, a risk factor for orbital invasion and recurrence has a low prevalence (<1%) [4, 27]. We do not have any confirmed cases even though we are convinced that we did not detect it in case 1 of Table 1, due to its recurrences and its evolution despite the treatments carried out, ending with an enlarged exenteration to obtain free margins, progressing and invading neighboring cavities until it led to the patient's death. In cases of PNI, we must clinically suspect it, since an MRI can help confirm it but cannot rule it out, and there may be cases with symptoms, but without radiological evidence [28].

We have found a higher incidence of tumors located in the medial canthus (66.6%); similar to what has been reported in other works (56.2% in Leibovitch et al. [8], 90% in Madge et al. [9], 62.5% in González et al. [6], and 87.5% in Tong et al. [11]. Tumors located in this area and in the lateral canthal area present a greater propensity to recurrence and orbital invasion, as has also been indicated by other authors [8, 25, 29–31], since the proximity of the skin to the periosteum in the medial and lateral canthal area is a factor that can predispose to orbital invasion.

#### 4.1.1. Clinical and Radiological Characteristics

Clinical signs that suggest orbital invasion have been identified, such as limitation of ocular motility, displacement of the globe due to mass effect, eyelid immobility, fixation of the tumor to the orbital rim, and sensory changes [5, 8, 9, 11, 32, 33]. We found, as the most frequent sign, a visible or palpable mass in 91.6% of the patients, similar to what was found by Madge et al. [9] (90%) or by Leibovitch et al. [8] (100%), and sometimes fixed to bone (10/24) (41.6%), in addition to eyelid retraction or immobility. We observed the alteration of ocular motility and the displacement of the globe in 50% and 33% of the cases, respectively, higher than what was observed in other works (30% and 17% by Leibovitch et al. [8] and 20% and 5% by Madge et al. [9], coinciding in cases with greater orbital invasion. These data indicate that orbital invasion is not always evident, and may be clinically silent, and therefore we must be alert in patients with risk factors, and maintain a high degree of suspicion and close follow-up [8, 10, 31].

Radiological test are necessary to verify orbital invasion in suspected cases. Most of the patients in our study (22/24) had a radiological test (CT or MRI) to check the orbital invasion and its location. In 75% of our cases, it was possible to verify the orbital invasion and its location, compared to 66.6% of cases reported by Madge et al. [9]. In 4 of our patients who had been previously operated on, radiological doubts arose regarding whether the invasive tissue was result of a recurrence or fibrous tissue secondary to surgery. Although in 2 of the oldest cases we were unable to verify the radiological evidence, we had confirmation of the report from their hospitals. CT with bony windows is the best test to rule out erosion of the bone walls, and MRI is the best test to detect soft tissue or nerve involvement [8, 28]. In our study, bone erosion was detected in 29.1% of the cases, similar to the 20.6% reported by Leibovitch et al. [8] or 27% by Howard et al. [12]. The clinical and radiological characteristics of the infiltration of the soft tissues that we observed in our work, like what was observed by other authors [8, 11], were mostly an irregular mass with increased uptake, which can invade the lacrimal or ethmoid fossa, the orbital floor, and can infiltrate the extraocular muscles and sclera, or invade deeper planes of the orbital space.

### 4.2. Discussion of Therapeutic Options

#### 4.2.1. Surgery

Surgery is the first line of treatment in cases of periocular BCC, whether it is wide surgical excision or Mohs surgery with frozen section margin control [1].

*(1) Orbital Exenteration*. Orbital exenteration was, until a few years ago, the main and only option to cure these tumors when they invaded the orbit [8, 12, 34–36]. Although it continues to be a useful treatment option, in recent years new therapeutic options have been tested to preserve the globe and vision, avoiding exenteration or extensive surgeries that result in great facial deformity or visual functional disability [9, 13, 15]. We think, like other authors [37], that if orbital exenteration is necessary, tumor eradication should be the primary criterion over any aesthetic or functional consideration. We have performed orbital exenteration in 9/24 cases (37.5%), in 7 of them enlarged with bone walls, a lower percentage of than those performed by Leibovitch et al. [8] (75%). The orbital exenterations that we performed out of the total number of periocular carcinomas surgically treated in the period studied represent 1.84% (9/487), the same as Wong et al. [30] (1.6%) and a lower percentage than that reported by Payne et al. [38] (3.6%), Perlman and Hornblass [39] (3.8%), and Howard et al. [12] (2.5%). It should be noted that the majority of these exenterations (6/9) were performed before 2016, and since that year, we have reduced their prevalence and attempted surgical treatments preserving the globe in most cases, adding treatment with vismodegib in some of them, as observed by other authors since the approval of this treatment by the US Food and Drug Administration (FDA) [40].

Exenteration was the technique we chose in cases called highly aggressive by Gerring et al. [41], that is, when the tumor infiltrated the periorbit, the sclera, the extraocular muscles, the retrobulbar fat, or the paranasal sinuses, and in when the prognosis and progression could be fatal and there were no other possible effective treatments [34, 42]. The purpose of choosing exenteration was to eliminate all the tissue infiltrated by the tumor and obtain free margins, as has been indicated by other authors [41]. In all the cases in which we performed total exenteration, we removed all the tissue from the orbital rim, including the periorbit, preserving, in some cases, the skin of the eyelids and using them in the subsequent reconstruction, the same as other authors [34].

When bone removal is necessary, ethmoidectomy or maxillectomy is usually performed, called extended orbital exenteration [43] in most cases included in this study. In 2/9 (22.2%) cases of exenteration, it was not possible to achieve a free margin due to tissue infiltration beyond the bone walls, which is a lower percentage than that reported in other studies (28–47%) [14, 44, 45]. RT (because vismodegib was not yet available) or vismodegib were added as an adjuvant treatment.

Some authors have indicated that RT has not been shown to improve the recurrence or survival rate after exenteration [10, 41], and it was not the adjuvant treatment of choice in our cases. The primary purpose of exenteration is to obtain negative margins, and not obtaining them is a poor prognostic factor, as some authors have pointed out [41], although this has not been demonstrated in some series [14, 44]. The published recurrence rate after exenteration ranges from 2.8 to 28%, which may be lower than with conservative surgery plus RT [5]. In our study, we found a recurrence rate after exenteration of 11.1% (1/9), within the published range, although higher than that reported in other series (2.8% in Leibovitch et al. [8] and 4.5% in Gerring et al. [41]. Survival after exenteration in our study is 88.8% at 4.66 years (4 years and 9 months, mean follow-up after exenteration), somewhat higher than in other studies (Rahman et al. [14] 75% and Hoffman et al. [46] 63% at 5 years, Gerring et al. [41] 78% at 2 years), although the deaths of some patients in the study by Rahman et al. [14] were shown to be unrelated to the tumor.

In all cases, after orbital exenteration, we covered the cavity with a transposition of temporalis muscle flap using a lateral transorbital approach, as described by Bhattacharjee et al. [47], preserving skin from the eyelid or with a skin graft of other donor areas to cover the skin defect, without further complications during follow-up. Mohr and Esser [37] previously suggested that by covering the cavity with soft tissues, without leaving spontaneous granulation, complications such as sinuorbital fistulae, or more serious complications such as meningoencephalitis, brain abscess or postradiation osteonecrosis, were reduced. In addition, the healing and recovery time is much faster since spontaneous granulation sometimes exceeds 3-4 months [48]. It has also been suggested that the use of split-thickness skin grafts to cover the orbit may have the advantage of not impairing the detection of a recurrence [49]. However, we believe that the risk of masking a possible local recurrence of the tumor by covering the orbit with soft tissue is small, since the risk of recurrence after exenteration itself has been shown to be low.

We solved the hollowing in the temporal fossa resulting from transposition of the temporal muscle with the placement of a high-density porous polyethylene implant that we fixed with titanium screws, achieving a good cosmetic result, as was also reported by other authors [50, 51].

An oculofacial epithesis can be placed over the orbital cavity, although most of our patients rejected it considering the final cosmetic result acceptable, as was reported in other studies [48, 52–54] (Figure 6(d)).

*(2) Globe-Sparing Surgery*. In half of the cases in our work (12/24), the CCB infiltrated only the anterior orbital space; therefore, it was decided to intervene by nonexenteration surgery, controlling margins and preserving the ocular globe, a technique with which Madge et al. [9] obtained good results with 5% recurrence rates and 90% survival (no recurrences in our study). Like Ho et al. [55], we performed excision of the tumor with the non-Mohs rapid paraffin technique in most cases, and reconstruction a few days later once the histological results were obtained. In 25% of the cases where we did not obtain free margins with this technique (3/12), we did not think it was possible to perform further excisions without risk of injuring the eyeball, muscles, or important orbital structures, and we decided to reconstruct the defect and apply subsequent treatment with vismodegib for several months, as it had already been shown to be effective in other cases after surgery [17, 56]. Mohs micrographic surgery is not the recommended technique for these tumors that infiltrate the orbit, since it is difficult to obtain orientation of the orbital soft tissue pieces, as well as being expensive and time-consuming, and with the technique with a standard frozen section the risk of false negatives is higher [1], although it was used in the oldest cases. The paraffin section technique is the technique that we used most frequently in our work and is the best for deep margin control when the tumor may be adjacent to the orbital fat [1, 5, 8, 9].

Complications derived from this treatment were as expected, epiphora being the most frequent (66.6%), as observed by Madge et al. [9] (75%), although we had fewer cases of postoperative muscle restriction. This could be explained because in some of the cases with orbital invasion and extraocular muscle involvement we decided to perform exenteration, and in those in which we decided to be more conservative but were unable to completely remove the tumor by surgery, subsequent treatment with vismodegib was instituted to remove tumor remnants without altering the conjunctiva, sclera or muscles. Finally, the rate of diplopia (16.6%) was very similar to that observed by these authors (15%). In our work, we do not include the interventions carried out to resolve this type of complications.

#### 4.2.2. Radiotherapy (RT)

RT, as the only treatment or as an adjuvant treatment after surgery, is a therapeutic option that was rarely used in our series. Although it can be a useful treatment in nonoperable cases, with recurrence rates of 25% [8, 35], patients have accepted the consequences of surgery, and we have evaluated other adjuvant treatment options after surgery, given the potential secondary effects on the ocular surface and vision, from xerophthalmia, cataract formation, neovascular glaucoma, retinopathy, optic neuritis to blindness [57]. Complications due to RT were observed in up to 25% of the cases in the series by Leibovitch et al. [8], in the periorbital region and in the eyeball. In addition to the complications that can threaten vision, it has also been pointed out in other works that it can threaten the recovery of the anophthalmic cavity after exenteration [8, 35, 57–59]. In this study, we operated on patients with recurrences and orbital invasion, previously treated with RT, with subsequent reconstruction being more difficult due to the changes in the skin and ocular surface produced by radiation, as also pointed out by Rene [60].

#### 4.2.3. Vismodegib for Advanced Periocular BCC

Sporadic genetic susceptibility mutations are involved in BCC pathogenesis. These mutations most commonly alter the Hh signaling pathway, which is an essential pathway for normal embryonic development, but also in the adult for the maintenance and regeneration functions of the skin and stem cells in the adult [61]. Molecular and genetic studies have shown that there is an altered signaling in the Hh pathway that leads to uncontrolled cell proliferation in basal cell carcinoma. Normally, this pathway is inhibited by the PATCH 1 gene, but when its activity is deficient due to mutations, a membrane protein called smoothing (SMO) is activated, which in turn activates the Hh pathway and ultimately causes tumorigenesis, BCC development, and progression. Vismodegib selectively binds to SMO, inhibiting Hh pathway activation and tumor cascade proliferation [40]. It was approved in 2012 by the FDA, by the European Medicine Agency (EMA) in 2013, and in 2016 authorized by the Ministry of Health of our country for financing through our National Health System for use in locally advanced BCC tumors and in metastatic tumors, where surgical treatment is no longer possible or RT treatment is not sufficient [1, 62–64], being well tolerated and demonstrating efficacy in around 50% of cases [65–67]. There are more and more studies showing a favorable clinical response in the treatment of BCC with vismodegib, in the periocular region or in any part of the body and of any histological type, even in basosquamous carcinoma [15, 65, 68, 69]. This also leads to an improvement in the emotional stress caused by the tumor in these patients [2]. In recent years, its use has been tested in advanced tumors with orbital invasion, in isolated cases or in case series, with a complete response to treatment between 25% and 87.5% [6, 40, 56, 66–71]. Vismodegib has not only been used as a sole treatment but also combined with surgery, as off-label use, either as a prior neoadjuvant or adjuvant after surgery [6, 15, 17, 56, 59, 65–67, 69, 71, 72].

We have not observed recurrence in our cases treated with vismodegib, as sole treatment or after surgery, preserving the ocular globe, or after exenteration; therefore, we can compare our results with other series in which vismodegib was used combined with surgery [6, 15, 17, 56], either before or after it, finding the same results.

It has been suggested that single treatments or for long-term treatments with vismodegib may influence the development of primary or secondary resistance to the drug [73, 74] and that its combination with surgery may be useful to counteract these effects [6]. We have not found any case of resistance to treatment in our study, despite the duration of more than 58 months in one of our patients.

Although in this study we used vismodegib postoperatively to eliminate residual tumor when free and clear margins were not achieved, its preoperative use also seems to be a useful option, as suggested by other authors [6, 15], to achieve tumor cytoreduction and perform definitive tumor excision as soon as possible to minimize the risk of recurrence through less deforming surgical reconstruction and globe preservation [75]. As some authors have pointed out, it must be taken into account that after neoadjuvant treatment the size of the tumor is reduced, but islands of tumor that are not seen macroscopically may remain; therefore, surgery may not fully encompass the tumor [15, 20, 76]. In these cases, some authors have suggested that it is advisable to follow up for at least 5 years in case there are recurrences and to perform map biopsies during this period [1, 15, 77]. The mean follow-up in our cases of surgery combined with vismodegib was 47.3 months (range 36–62 months), higher than the mean follow-up in other series such as Sagiv et al. [15] (18 months) or González et al. [6] (12.4 months).

*(1) Duration of Treatment*. Although the efficacy of treatment with vismodegib has been demonstrated, including in our study, we have doubts about the duration of treatment, as do other authors [59]. One of our patients with neoadjuvant treatment for 12 months presented a complete response and no recurrences during follow-up. Another patient with Gorlin's syndrome, who underwent numerous surgeries and RT previously, after 58 months of treatment with vismodegib after the last surgery preserving the eyeball, has not presented any recurrence. The patient continues to display tolerance to treatment and has not developed resistance (Figure 5(f)). Two of our patients, after surgery without free margins, were treated for 10 months with vismodegib, with no recurrences (Figure 5(i)) during follow-up. In another patient, an improvement was verified after 8 weeks of treatment, although it had to be suspended after the third month of treatment, as oncological treatment for lung cancer had to be started, and there are insufficient data on vismodegib's safety and interactions in combination with other cancer treatments. As in our study, a favorable response has also been seen in short periods of treatment, after 11 weeks [66], or complete responses after 3 months of treatment [15]. The efficacy of the treatment depends on its duration, and according to Sekulik et al. [65], the time in which maximum tumor reduction is estimated is 5.5–6.7 months, similar to that observed in other series (4.8 months in González et al. [6]). Although there is no agreement on the minimum recommended duration of treatment, some authors have suggested that it requires a minimum of 3 months of treatment [78]. We think, like Sagiv et al. [15], that patient-dependent factors such as age or immune status, and other tumor-dependent factors, such as size, extension, or histological type, may influence the variation in duration of the treatment.

*(2) Side Effects of Treatment*. Most of the patients that were treated with vismodegib in our study (5/7), alone or combined with surgery, experienced side effects. These were mild and bearable in all cases except in an elderly patient, 95 years old, who had to stop treatment. In no other case have we interrupted treatment due to side effects, even after 58 months of treatment. The most frequently observed side effects are muscle spasms (71.4%), dysgeusia (57.1%), hair loss (50%), gastric discomfort, and weight loss (50%), which coincides with what other works have reported [15, 65, 67, 68, 70, 79]. Interruptions in treatment are possible if we need to increase its tolerability and we want to avoid its abandonment. In some studies, up to 30% of patients have discontinued treatment due to adverse effects, and although most are reversible, some cases of permanent alopecia have been seen [79]. Different ways of reducing the dose during treatment have been proposed, with the aim of increasing patient's compliance and adherence to the treatment [80–82]. Becker et al. [83] verified that a decrease in the frequency of the administered doses increases tolerability and decreases side effects, without diminishing the effectiveness of the treatment. Also, Sekulic et al. [65] verified this in the ERIVANCE study, concluding that treatment interruptions (on-off regime), even of several weeks, did not influence the duration or the response to treatment, but also allowed longer treatments, as we have also been able to verify in our case with the longest evolution treatment, with good tolerance and no recurrence despite the interruptions. Although this should be studied further to help find the optimal duration of treatment, there is consensus regarding the value of introducing vismodegib interruptions in the recommended treatment strategies in patients with advanced BCC [80]. We have not observed, among our cases treated with vismodegib, the appearance of any epidermoid (squamous) lineage tumor. Although some references can be found in the literature [84], an increase in development of squamous cell carcinoma has been ruled out in patients treated with vismodegib [85], but a change in histology to a metatypical BCC or even basosquamous may occur [64].

Like other authors [6, 67], we believe that in cases of advanced BCC with orbital invasion and involvement of essential ocular structures such as muscles, sclera, and lacrimal apparatus, the primary goal of treatment should be to preserve the globe and vision, and the data obtained suggest that vismodegib, whether or not combined with surgery (without exenteration), can be very effective for this purpose.

### 4.3. Limitations

This study aims to analyze the therapeutic options for periocular BCC with orbital invasion and the management of new therapies that can change our current perspective, when it comes to indicate mutilating or highly disfiguring surgeries. The retrospective nature of our study is one of the limitations. The use of new therapies, such as vismodegib, is promising but we still have doubts about how to use it to maximize its effectiveness. Originally, it was indicated in cases in which surgery was impossible, but we are recently discovering other ways of using it. Good results are being obtained by using it in combination with globe-sparing surgery, but we are still in the early stages of these studies. In our work, we used this medication after surgery, in cases, where we did not totally eliminate the tumor, and in one of them eliminating the eyeball to prevent its progression to neighboring cavities, but there are few similar studies where we can compare the results. We need more studies and more patients to be able to compare the results. The follow-up time of these patients in our work is longer than in other similar studies, but it is still insufficient. The main difficulty found in the studies published to date, such as ours, is that the drug has been approved for use for a relatively short time, which has limited the grouping of large case series and sufficient follow-up time, although we hope that in future papers this will change.

## 5. Conclusions

Periocular BCC can invade the orbit and cause great morbidity, so complete histologic excision and follow-up for several years is desirable in all cases. We must be vigilant for tumors with risk factors for orbital invasion, as they can often be clinically silent.

If orbital invasion is identified, surgical treatment remains an important option, as it has high cure rates but can be very disfiguring, mutilating, and disabling if vision is lost. In recent years, targeted therapies such as HPI have been developed that we can effectively use instead of or in combination with surgery to cure the most severe cases. These new therapies have led us to change our perspective regarding every case of periocular BCC with orbital invasion. They allow us to propose a more conservative therapy than exenteration, which is safe, effective, and preserves the globe without compromising visual acuity.

## Figures and Tables

**Table 1:** Baseline demographic and clinical characteristics.

Patient	Age/gender	Histologic subtype	Localization	Primary/recurrent	TNM	Treatment (months)	Margins	Adjuvant therapy (months)	Clinical response	Follow-up(months)	Final outcome
1	42 F	Infiltrative	MC	Recurrent	T4a	Exenteration	Positive	RT	Progression	44	Exitus
2	78 M	Infiltrative	MC	Primary	T4a	Conservative surgery	Negative		CR	60	NED
3	60 F	Infiltrative	MC	Primary	T4a	Conservative surgery	Negative		CR	64	NED
4	75 M	Infiltrative	LC + UE	Recurrent	T4b	Exenteration	Negative		CR	76	NED
5	84 F	Infiltrative	MC + LE	Recurrent	T4b	Exenteration	Negative		CR	72	Exitus^*∗*^
6	85 F	Infiltrative	LE	Recurrent	T4b	Exenteration	Negative		CR	70	Exitus^*∗*^
7	82 M	Infiltrative	LE	Recurrent	T4a	Exenteration	Negative		CR	60	Exitus^*∗*^
8	91 F	Basosquamous	MC + UE + LE + LC	Recurrent	T4a	Exenteration	Negative		CR	36	Exitus^*∗*^
9	77 F	Infiltrative	MC	Recurrent	T4a	Vismodegib (12)			CR	48	NED
10	84 M	Infiltrative	LE	Recurrent	T4a	Conservative surgery	Negative		CR	41	Exitus^*∗*^
11	70 M	Infiltrative	MC	Recurrent	T4b	Exenteration	Negative		CR	60	NED
12	64 M	Infiltrative	MC	Primary	T4a	Conservative surgery	Negative		CR	72	NED
13	72 M	Infiltrative	MC	Primary	T4a	Conservative surgery	Negative		CR	59	NED
14	82 F	Infiltrative	MC	Recurrent	T4a	Exenteration	Negative		CR	44	NED
15	81 F	Infiltrative	MC	Recurrent	T4b	Exenteration	Positive	Vismodegib (8)	CR	42	NED
16	45 M	Infiltrative	MC	Recurrent	T4a	Conservative surgery	Positive	Vismodegib (58)	CR	62	NED
17	57 M	Infiltrative	MC	Recurrent	T4a	Conservative surgery	Negative		CR	60	NED
18	78 F	Infiltrative	MC	Recurrent	T4a	Conservative surgery	Negative		CR	62	NED
19	52 M	Infiltrative	LC + LE	Primary	T4a	Conservative surgery	Negative		CR	54	NED
20	54 M	Infiltrative	LC + LE	Primary	T4a	Conservative surgery	Negative		CR	62	NED
21	79 F	Infiltrative	MC	Recurrent	T4a	Conservative surgery	Positive	Vismodegib (10)	CR	44	NED
22	67 M	Infiltrative	MC	Recurrent	T4a	Vismodegib (3)				12	Exitus^*∗∗*^
23	95 F	Infiltrative	MC	Primary	T4a	Vismodegib (1)				8	Exitus^*∗*^
24	72 M	Micronodular	MC	Recurrent	T4a	Conservative surgery	Positive	Vismodegib (10)	CR	36	NED

F: female, M: male, TNM: staging tumor node metastasis [19], MC: medial canthus, LC: lateral canthus, UE: upper eyelid, LE: lower eyelid, ^*∗*^not due to cancer, ^*∗∗*^due to lung cancer, RT: radiotherapy, CR: complete remission, NED: no evidence of disease.

## Data Availability

The data used to support the findings of this study are available from the corresponding author upon request.
